# An Exuberant Calcifying Epithelial Odontogenic Tumor in the Posterior Maxilla: A Case Report

**DOI:** 10.4317/jced.62701

**Published:** 2025-05-01

**Authors:** Rodrigo Silva, Carolina Ruppel, Ramon Cesar Godoy Gonçalves, Roberto de Oliveira Jabur, Ana Luiza Oliveira Corrêa Roza, Pablo Agustin Vargas, Marcelo Carlos Bortoluzzi

**Affiliations:** 1 Oral and Maxillofacial Surgery Residency Program at University Hospital of Campos Gerais (HUCG); 2 Dentistry Post-Graduate Program, State University of Ponta Grossa (UEPG), Ponta Grossa, Brazil; 3 Oral and Maxillofacial Surgery Residency Program at University Hospital of Campos Gerais (HUCG), Ponta Grossa, Brazil; 4 School of Dentistry, State University of Ponta Grossa (UEPG), Ponta Grossa, Brazil; 5 Department of Oral Diagnosis, Piracicaba Dental School, University of Campinas (UNICAMP), Piracicaba, Brazil; 6 Department of Oral Diagnosis, Piracicaba Dental School, University of Campinas (UNICAMP), Piracicaba, Brazil; 7 School of Dentistry, Dentistry Post-Graduate Program, Oral and Maxillofacial Surgery Residency Program at University Hospital of Cam pos Gerais (HUCG), State University of Ponta Grossa (UEPG), Ponta Grossa, Brazil

## Abstract

This case report presents a rare instance of a Calcifying Epithelial Odontogenic Tumor (CEOT) in the maxilla of a 52-year-old male, characterized by a painless, growing mass and facial asymmetry. Imaging revealed extensive involvement of the maxillary sinus, nasal cavity, pterygoid plate, and infraorbital margin. Histopathological analysis confirmed the diagnosis, showing polyhedral odontogenic epithelium with amyloid-like material. The patient underwent conservative surgical excision, preserving function and aesthetics, with no recurrence observed at 16 months postoperatively. This case underscores the importance of a multidisciplinary approach in managing CEOT, particularly in complex anatomical regions, to achieve favorable outcomes. Long-term follow-up remains essential.

** Key words:**Calcifying Epithelial Odontogenic; Pindborg tumor; CEOT; maxilla; odontogenic tumors.

## Introduction

According to the 2022 WHO classification of head and neck tumors, the Calcifying Epithelial Odontogenic Tumor (CEOT), also known as the Pindborg tumor, is a rare benign odontogenic epithelial tumor, with uncertain pathogenesis, that accounts for about 1% to 1.7% of all odontogenic tumors (1-6). However, it may exhibit locally aggressive behavior with the potential to invade surrounding normal jawbone tissues, and there have been occasional reports of malignant transformation (1-7).

CEOT typically presents as a slow-growing, painless swelling which affects both sexes equally, with a wide age range with peak incidence occurring in the third and fourth decades of life (1,5,6). It can manifest in two forms: intraosseous (central) or extraosseous (peripheral). The intraosseous variant (presentation is seen in over 80% of cases) is more frequently observed in the posterior region of the mandible, whereas the extraosseous variant is more commonly found in the anterior gingiva, where it may appear as a sessile mass and is also capable of causing destruction of the underlying bone (1,2,4). The mandibular premolar and molar regions are the most common sites of involvement, accounting for 60% to 68% of cases, while the maxilla is less frequently affected with about half of all CEOT cases associated with an impacted tooth (1,2). The radiographic presentation of CEOT depends on the stage of development and may presents as a unilocular or multilocular radiolucent lesion, often containing varying degrees of radiopacity due to dystrophic calcifications. The lesion’s borders may range from well-defined to ill-defined, depending on the tumor’s aggressiveness and degree of cortical bone infiltration (2,5,8).

Histopathological findings reveal sheets, islands, and cords of polyhedral epithelial cells embedded within a fibrous connective tissue stroma, characterized by prominent intercellular bridges, cellular pleomorphism, and hyperchromatism. A hallmark feature of CEOT is the presence of amyloid-like material, which appears as an eosinophilic, homogeneous, and amorphous deposit, identified as odontogenic ameloblast-associated protein. This amyloid material often undergoes calcification, forming concentric calcified structures known as Liesegang rings (1,5,6), nevertheless, the tumor present histopathological variants which may produce differences in the tumor biological behavior (2-4). CEOT may occasionally infiltrate the medullary bone and exhibit worrisome features that may cause confusion with malignancy (9).

Published cases of CEOT are limited, and opinions on its biologic behavior vary in the literature. Some authors (8,10) suggest that CEOT behaves aggressively, similar to ameloblastoma, and recommend comparable surgical approaches, such as marginal or segmental resection with at least 1.0 cm margins. However, there is evidence which suggests that CEOT is less aggressive than ameloblastoma, favoring conservative resection with narrow margins of healthy bone (1,2,6). Despite this approach, recurrence rates have been reported to range from 10% to 22% (2,5,6,8,10).

This report presents an uncommon case of CEOT in the maxilla with extensive involvement of the maxillary sinus, nasal cavity, pterygoid plate, and infraorbital margin in a male patient.

## Case Report

Ethical Considerations and Confidentiality: The patient provided written informed consent for publication, and the case study was reviewed and approved by the Institutional Human Research Ethics Committee (approval number 7.251.599).

**Figure 1:**
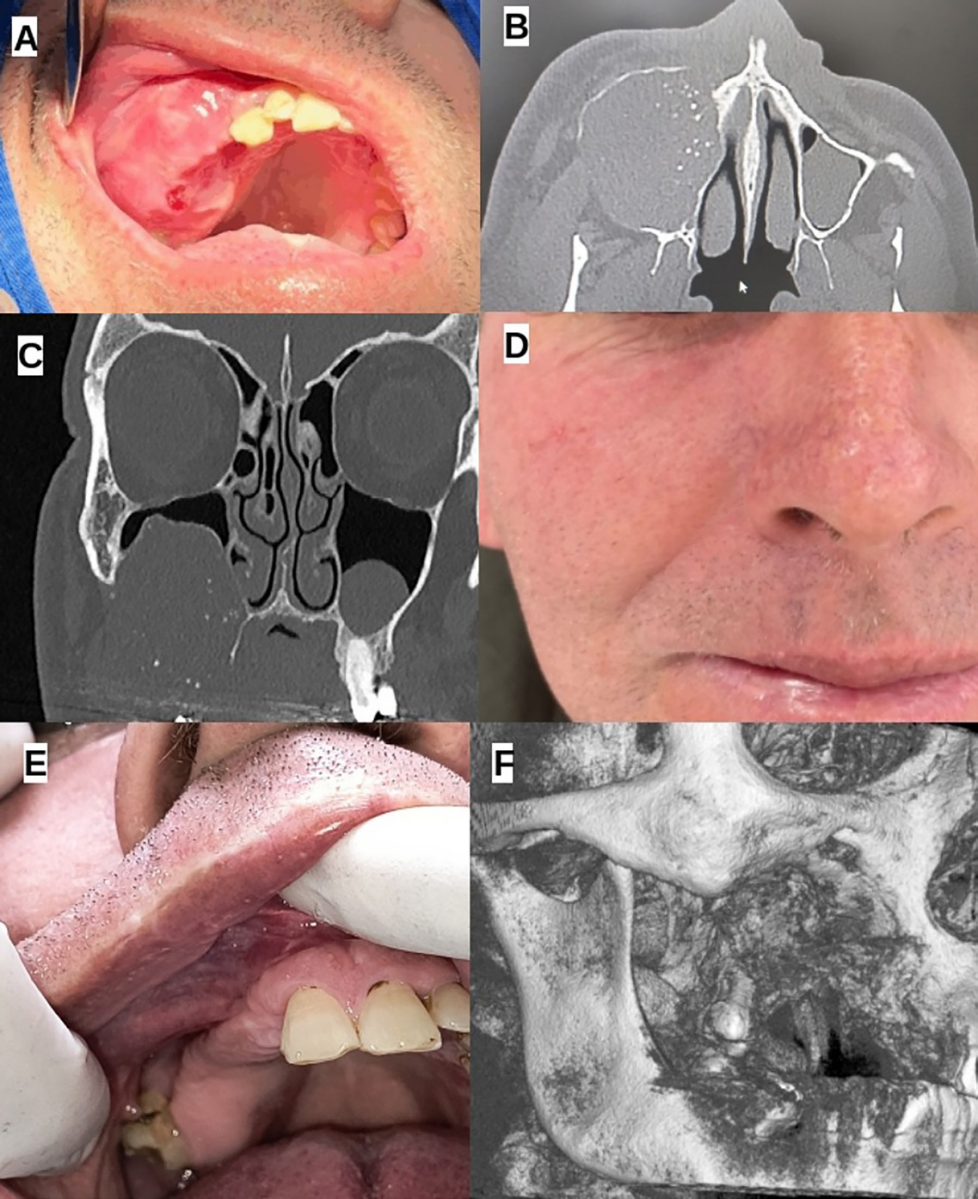
Clinical and imaging findings of a calcifying epithelial odontogenic tumor (CEOT) in the right maxilla. (A) Intraoral clinical view showing a firm, well-defined swelling in the right maxillary region. (B) Axial computed tomography (CT) scan demonstrating an expansive lesion with cortical expansion, thinning, bony destruction, and internal calcifications involving the right maxillary sinus. (C) Coronal CT scan illustrating the tumor’s superior extension towards the orbital floor without clear orbital invasion, as well as its involvement of the alveolar ridge and hard palate. (D) Extra-oral view showing no significant facial scarring or asymmetry following surgical intervention. (E) Intraoral examination revealing a well-healed surgical site without signs of recurrence. (F) 3D reconstructed CT image illustrating bone remodeling and the absence of tumor recurrence.

Clinical History and Findings: A 52-year-old Caucasian male sought care due to an asymptomatic, intraoral growing mass. The patient had previously been treated by a general dentist, who initially misdiagnosed the lesion as a periodontal abscess due to dental mobility in the affected area. Consequently, the patient underwent dental extractions in an attempt to treat the presumed abscess. Upon clinical examination, the patient presented with a firm, well-defined, painless swelling in the right maxillary region and facial asymmetry which had been present for approximately six months. Figure 1A-C illustrates the preoperative clinical and imaging findings of the calcifying epithelial odontogenic tumor (CEOT), showing the tumor’s extent and involvement of the maxillary sinus, nasal cavity, and infraorbital margin, while Figure 1D-F demonstrates the postoperative outcomes, highlighting the absence of recurrence and favorable healing at 16 months follow-up.

Computed Tomography Imaging Findings: The computed tomography (CT) imaging findings shows an expansive and aggressive lesion. The tumor demonstrates significant involvement of the right maxillary sinus, with cortical expansion, thinning, and areas of bony destruction and with the presence of internal calcifications (Fig. 1B). The lesion extends superiorly toward the orbital floor, without clear signs of invasion into the orbital cavity (Fig. 1C). Inferiorly, the tumor involves the alveolar ridge and hard palate, with cortical erosion suggesting possible extension into the palatal soft tissues. Posteriorly, the lesion extends to the pterygoid region, indicating potential deep tissue involvement that may complicate surgical management. Additionally, the mass exerts an effect on the right nasal cavity, displacing the lateral nasal wall toward the left. While no gross invasion of the pterygoid plate or nasal mucosa is evident, the lesion’s close proximity to these structures raises concerns about possible secondary involvement. Given the tumor’s size, location, and associated bone remodeling, these findings suggest a locally aggressive yet well-contained lesion with no clear evidence of distant spread.

**Figure 2:**
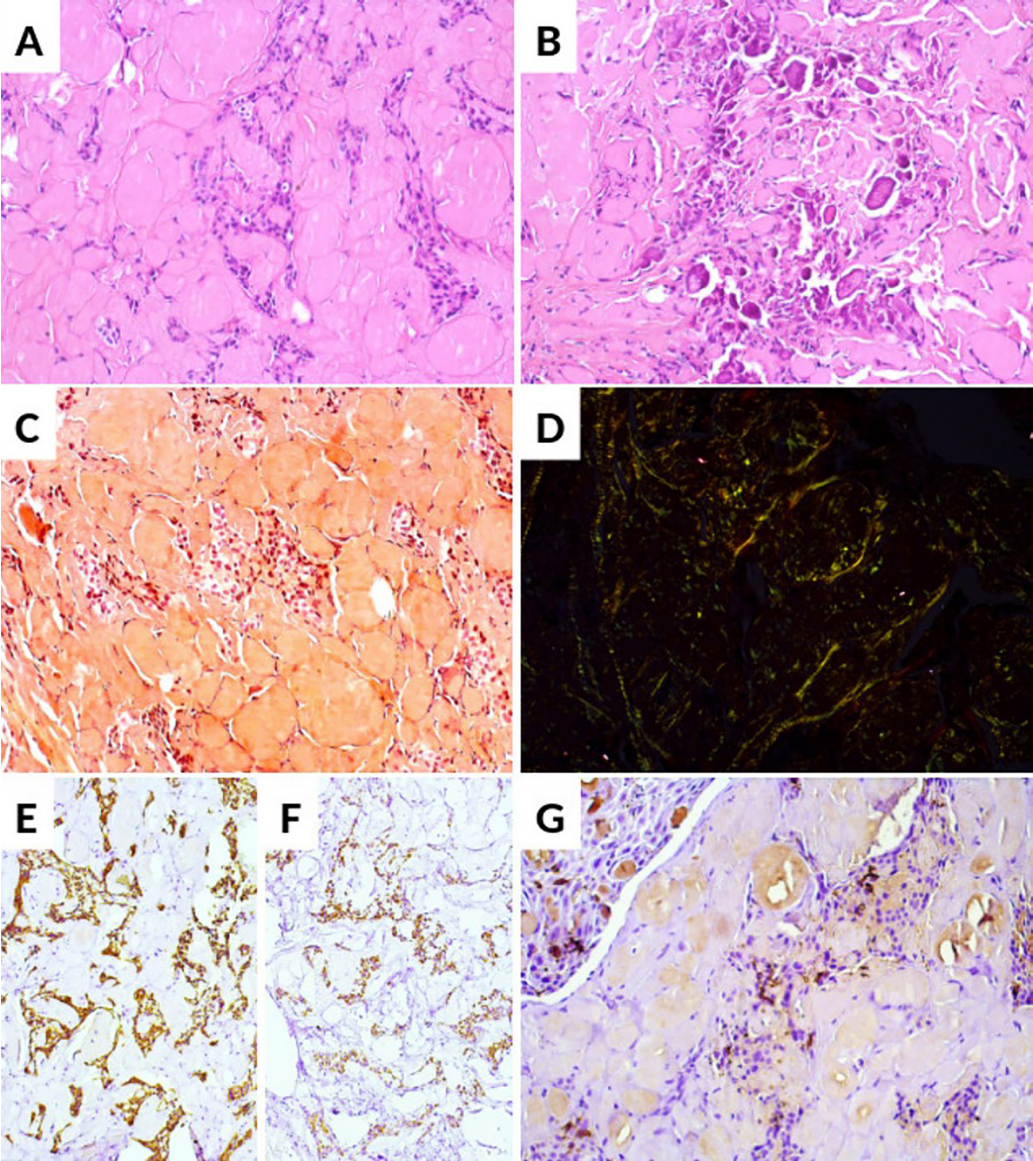
Histopathological and immunohistochemical findings of CEOT. (A, B) H&E staining showing focal areas of amyloid-like deposits undergoing calcification. (C) Congo red staining highlighting amyloid-like material. (D) Green birefringence under polarized light confirming amyloid deposition. (E, F) Immunohistochemical staining showing diffuse positivity for AE1/AE3 (E) and CK19 (F) in the epithelial islands. (G) CD1a immunostaining highlighting occasional Langerhans cells interspersed within the odontogenic epithelium.

An incisional biopsy was performed under local anesthesia and the tissue submitted for histologic analysis. Microscopic examination revealed a tumor comprised of multiple islands and strands of polyhedral odontogenic epithelium showing brightly eosinophilic cytoplasm and prominent intercellular bridges embedded in an amorphous eosinophilic material consistent with amyloid. In focal areas, the amyloid-like deposits underwent calcification (Fig. 2A-B). Congo red staining was positive and showed green birefringence under polarized light (Fig. 2C-D). The epithelial islands demonstrated diffuse positivity for AE1/AE3 and CK19, whereas CD1a highlighted occasional Langerhans cells intermingled within the odontogenic epithelium (Fig. 2E-G). The final diagnosis was CEOT.

Due to the extent of the lesion, an initial approach of enucleation and curettage was chosen. Surgical access was achieved using the Weber-Ferguson approach. The tumor was meticulously dissected from the surrounding structures and excised as a single specimen. Given the presence of a communication between the posterior maxillary sinus, the posterior border of the orbital cavity, and the skull base, a resorbable mesh was placed to facilitate secondary closure of the defect. Primary wound closure was achieved by carefully dissecting and mobilizing the oral mucosa. The procedure was completed without complications. The patient had an uneventful postoperative recovery and was discharged 48 hours after surgery.

Follow-up evaluations were scheduled every three months, incorporating detailed clinical examinations and computed tomography (CT) imaging. Throughout the follow-up period, the patient remained asymptomatic, with no signs of paresthesia or significant facial scarring due to the surgical access. At 16 months postoperatively, the patient remained disease-free, with no clinical or tomographic evidence of recurrence (Fig. 1D-F).

## Discussion

Calcifying Epithelial Odontogenic Tumor (CEOT) is a rare odontogenic neoplasm with locally aggressive behavior, often requiring extensive surgical management (1,6,8,10). Despite being classified as a benign tumor, CEOT demonstrates a capacity for cortical bone destruction and extension into adjacent anatomical structures, as observed in this case. The involvement of the maxillary sinus, nasal cavity, and infraorbital region presented a unique surgical challenge, as maxillary lesions tend to be more aggressive and infiltrative compared to mandibular cases (8). Given these characteristics, careful preoperative planning, including advanced imaging and 3D modeling, was essential for understanding tumor boundaries and ensuring complete resection while minimizing functional and esthetic compromise.

According to Li *et al*. (2025) (6), only approximately 430 cases of CEOT had been reported in the literature by 2023 and between those, very few cases have been reported in the maxilla with extension to the maxillary sinus (5). Due to this limited data and the lack of long-term outcome studies, optimal management strategies for CEOT remain unclear. As a result, surgical treatment should be tailored to each case, taking into account specific clinical and histopathological characteristics. Key factors influencing the surgical approach may include the tumor’s anatomic location, size, clinical behavior (e.g., cortical bone penetration, periosteal or muscle involvement, neural invasion), and histopathological features (e.g., neural or vascular infiltration, cellular atypia, presence of clear cells, and mitotic activity) (1,6,8,10).

While the optimal surgical management of CEOT remains a subject of debate, with some authors advocating conservative enucleation and curettage and others recommending segmental resection with safety margins to reduce recurrence risk, enucleation with curettage is generally reserved for very small, well-confined lesions8,10. In this case, given the well-demarcated nature of the tumor and the need to minimize functional and esthetic morbidity, a conservative surgical approach was chosen. This strategy allowed for complete excision of the lesion while preserving adjacent structures.

Histopathological analysis remains the gold standard for diagnosing CEOT, as its radiographic presentation can overlap with other benign and malignant odontogenic tumors. The presence of polyhedral epithelial cells, amyloid-like deposits, and Liesegang ring calcifications confirmed the diagnosis in this case. Some histopathological features, such as clear cell changes, increased mitotic activity, and nuclear pleomorphism, have been associated with a more aggressive clinical course or potential malignant transformation. Although these features were not observed in the present case, the tumor’s extensive involvement and cortical perforation reinforced the need for an extensive surgical approach and long period of follow-up.

Long-term follow-up is crucial due to CEOT’s potential for recurrence, reported in up to 22% of cases, as well as the risk of malignant transformation. Regular clinical and radiographic monitoring is necessary to detect any signs of relapse early and ensure timely intervention (4,5,7). Factors such as incomplete resection, lesion size, and anatomical location may also influence recurrence rates. In this case, meticulous resection and regular clinical and radiographic follow-up have ensured a favorable outcome, with no signs of recurrence at 16 months postoperatively.

## Data Availability

The datasets used and/or analyzed during the current study are available from the corresponding author.
